# Editorial: Advancements in equine pain management

**DOI:** 10.3389/fpain.2025.1547764

**Published:** 2025-02-05

**Authors:** Ludovica Chiavaccini, Rachel Anne Reed, Claudia Spadavecchia

**Affiliations:** ^1^ Department of Comparative, Diagnostic, and Population Medicine, College of Veterinary Medicine, University of Florida, Gainesville, FL, United States; ^2^ Department of Large Animal Medicine, College of Veterinary Medicine, University of Georgia, Athens, GA, United States; ^3^ Department of Clinical Veterinary Medicine, Anaesthesiology and Pain Therapy Section, Vetsuisse Faculty, University of Bern, Bern, Switzerland

**Keywords:** equine, pain, drug therapy, quantitative sensory testing (QST), clinical metrology index

**Editorial on the Research Topic**
Advancement in equine pain management

“Pain is a more terrible lord of mankind than even death itself.”—Albert Schweitzer. Pain is a global issue affecting humans and animals, and over the past decade, research into its biology, treatment, and prevention has flourished. However, progress in equine pain research has lagged behind its small animal counterpart. Painful conditions in horses are often overlooked by owners and practitioners, leading to inadequate recognition and management of pain (1–3). Accurate pain assessment is essential for ensuring appropriate analgesia in equine patients. Yet, studies reveal significant gaps in knowledge and action, even among confident horse owners in the UK and US (2). For example, while owners may recognize signs of colic (abdominal pain), their responses to emergencies are often inconsistent. Furthermore, equine pain recognition and treatment remain underexplored in low- and middle-income countries (Laleye et al.), where limited resources, inadequate training, cultural diversity, and language barriers contribute to animals not receiving basic pain treatment. The retrospective study by Laleye et al. demonstrated that delays in recognizing abdominal pain and referring horses for treatment increase mortality and hospital expenses, in Senegal.

Some of this discrepancy in pain recognition stems from the subtle and multifaceted expressions of pain in horses (3). Although several equine pain scales have been developed (4–9), their implementation in practice remains inconsistent. Factors such as lack of exposure, misinterpretation, or overinterpretation of these tools contribute to the gap in pain recognition and treatment (10). For example, Reed et al. showed that the residual effects of general anesthesia may affect the accuracy of facial expression-based pain scoring systems in the hours immediately following anesthetic recovery.

The inherent subjectivity of evaluating pain behavior is a challenge to achieving objective and quantitative pain assessment. A standardized scale for clinical and research applications could address these limitations. In the study of Nowak et al., specific behavioral indicators—such as weight shifting and unstable resting—appeared reliable tools for distinguishing between horses experiencing musculoskeletal pain and those that were pain-free. Severely painful horses displayed reduced feeding and resting behavior while standing, along with increased unstable resting. These findings align with previous research highlighting postural behaviors as dependable pain indicators, particularly in orthopedic conditions (11, 12). In this issue, Auer et al. refined an equine musculoskeletal pain scale (MPS) by integrating elements of the equine pain face (13, 14), posture, head–neck position, weight-bearing, and weight shifting. This updated MPS provides a comprehensive framework for assessing chronic orthopedic pain in horses and explores incorporating visual information into automated pain recognition systems, offering new avenues for introducing objectivity in pain assessment in veterinary medicine (15).

While subjective pain scales and objective gait analysis systems quantify lameness severity in horses (16, 17), quantitative sensory testing (QST) methods—widely used in human medicine to define pain phenotypes—remain underexplored in equine practice. Mechanisms such as impaired autonomic joint innervation, nociceptive fiber plasticity, and dysfunction of descending pain inhibitory pathways likely contribute to chronic pain persistence. Gisler et al. demonstrated the feasibility and reliability of periarticular pressure pain assessment in healthy horses' distal thoracic limb joints. Their findings showed good repeatability among researchers, suggesting that periarticular pressure mapping could be a valuable complementary diagnostic tool for evaluating and mapping orthopedic pain phenotypes in horses. Furthermore, QST devices hold potential for assessing disruptions in modulatory pathways associated with chronic pain, offering insights into peripheral and central sensitization. For instance, the lip twitch, causing pressure on the upper lip, has been hypothesized to activate opioidergic and non-opioidergic descending modulatory pathways in horses (18). Blum et al. supported this theory by showing that lip-twitch application increased nociceptive withdrawal reflex and thermal pain thresholds in healthy horses. Integrating QST methods and assessments of conditioned pain modulation into equine practice could advance understanding of chronic pain mechanisms, improving diagnostic and therapeutic approaches.

Opioids are integral to analgesic protocols due to their high potency and efficacy in treating different pain in human and veterinary medicine. Injectable µ-receptor opioid agonists such as morphine, hydromorphone and methadone are commonly used perioperatively in horses. However, concerns about excitation (19), decreased gastrointestinal motility (20–22), and the conflicting data on the analgesic efficacy of full mu opioids in horses with naturally occurring diseases (23) often deter their use. In this special issue, Paranjape et al. and Reed et al. explored the pharmacokinetics and pharmacodynamics of transdermal buprenorphine and fentanyl in horses. These studies demonstrated good tolerance and prolonged therapeutic plasma concentrations but variable efficacy in increasing thermal or mechanical thresholds. Haralambus et al. investigated the incidence of postoperative colic (PAC), reporting an overall rate of 15.1%. The study also examined opioid use, noting that intraoperative or short-term postoperative administration did not increase PAC rates. However, long-term administration (greater than 24 h) of morphine significantly raised the PAC incidence to 34% (*p* = 0.0038), whereas long-term butorphanol or methadone had no significant effect. These findings underscore the importance of cautious opioid selection in postoperative pain management and use of the lowest effective dose and frequency.

Given the limitations of prolonged opioid treatment, non-pharmacological interventional techniques may offer valuable alternatives for managing chronic pain in horses. In this issue, Amari et al. propose ultrasound-guided radiofrequency ablation of the palmar digital nerve as a potential treatment for horses with chronic distal forelimb lameness. Histopathological findings revealed consistent axonal degeneration, which, in clinical settings, would translate into effective management of chronic pain, as seen in human and veterinary literature.

In conclusion, the Special Issue of “*Advancements in equine pain management”* features 11 articles showcasing the latest progress in understanding and managing equine pain. The collection explores diverse topics, including diagnostic innovations, pharmacological advances, and cutting-edge interventional techniques. While addressing every facet of equine pain in a single issue is impossible, this selection emphasizes novel methodologies and interdisciplinary research to improve equine welfare. Authored by leading experts in the field, these articles provide a comprehensive and accessible overview of equine pain recognition and management, making them invaluable resources for researchers and clinicians. The contributions highlight advancements from foundational science to clinical applications, employing a “bench to bedside” approach that bridges research and practice. We hope this collection will provide an insightful reading and inspire further innovation and collaboration in the ongoing effort to improve the lives of equine patients across the globe.
